# Biochar Application Improves Eggplant Yield in Association with Changes in Soil Microbial Communities and Soil Multifunctionality

**DOI:** 10.3390/microorganisms14102139

**Published:** 2026-09-23

**Authors:** Fangtian Liu, Qijin Kang, Chunxiang Song, Yude Wu, Fu Li

**Affiliations:** College of Science, Jiamusi University, Jiamusi 154007, China; 13503699906@163.com (F.L.); 19503600219@163.com (Q.K.); scx09090718@163.com (C.S.); wyd9518@163.com (Y.W.)

**Keywords:** biochar, soil multifunctionality, microbial community, eggplant yield, mollisols

## Abstract

Biochar is widely used to improve soil quality and crop productivity; however, the relationships among biochar addition, soil multifunctionality (SMF), microbial communities, and crop yield remain unclear. A field experiment was conducted from May to September 2025 in a continuous eggplant (*Solanum melongena* L.) cropping system established on Mollisols. Four biochar application rates (0, 5.40, 6.75, and 8.10 Mg ha^−1^) were evaluated using a randomized complete block design with three replicates. Biochar addition significantly affected eggplant growth, selected photosynthetic traits, root development, and yield, with the most consistent positive responses observed at 6.75 Mg ha^−1^. Biochar also altered soil physicochemical and biological properties, with these effects being more pronounced in the topsoil and at the fruiting stage. SMF was positively correlated with yield, and this relationship was stronger at the fruiting stage than at the flowering stage. Random forest analysis showed that yield was strongly associated with soil biological properties, particularly extracellular enzyme activities involved in carbon, nitrogen, and phosphorus acquisition. Structural equation modeling further examined depth-specific statistical associations among biochar addition, microbial communities, SMF, and yield; topsoil bacterial communities and subsoil fungal communities showed the strongest associations with SMF. Overall, the application of 6.75 Mg ha^−1^ biochar produced the most consistent improvements in SMF and eggplant productivity.

## 1. Introduction

Eggplant (*Solanum melongena* L.) is an important vegetable crop in intensive agricultural systems [1,2], but its sustainable production is often constrained by soil degradation associated with continuous monocropping [3]. Continuous eggplant monocropping can cause soil compaction, nutrient imbalances, acidification, reduced microbial activity, and yield losses [4]. These problems are particularly pronounced in high-input vegetable systems, where frequent fertilization and continuous cropping accelerate the deterioration of soil physicochemical and biological properties [4,5]. Therefore, developing effective soil amendments to improve soil quality and sustain crop productivity is essential for long-term eggplant production.

Biochar, a carbon-rich material produced by pyrolysis of biomass under oxygen-limited conditions, has been widely used as a soil amendment [6,7]. Its porous structure, large surface area, and recalcitrant carbon content enable biochar to reduce soil bulk density, improve aeration and water retention, increase soil organic carbon (SOC) content, and create favorable microsites for microbial colonization [7,8]. Biochar can also increase soil pH and regulate nutrient retention and availability, thereby improving conditions for plant growth [9]. However, its effects on crop yield are often inconsistent and depend on factors such as biochar type, application rate, soil properties, crop species, and management practices [10]. Moderate application rates typically enhance soil fertility and productivity, whereas excessive application may produce diminishing returns or even reduce nutrient availability through increased nutrient adsorption or disrupting microbial processes [11,12]. Thus, a critical question is not only whether biochar increases crop yield, but also which soil properties and biological processes underpin its effects on crop productivity.

Previous studies have largely attributed biochar-induced yield increases to improvements in soil structure, pH, nutrient retention, and organic carbon, yet these changes may not fully explain crop responses [12,13]. Crop productivity depends not only on nutrient pools but also on biological processes that transform soil organic matter into plant-available nutrients [14]. Soil microbial biomass and extracellular enzyme activities play central roles in these processes [15]. For example, β-glucosidase mediates carbon cycling, acid phosphatase facilitates organic phosphorus mineralization, and N-acetyl-β-glucosaminidase contributes to organic nitrogen turnover [16]. Therefore, biochar-induced yield responses may be more closely associated with changes in soil biological properties and nutrient-cycling processes than with increases in soil nutrient stocks alone [17]. However, the extent to which biological properties mediate biochar-induced improvements in soil multifunctionality (SMF) and eggplant yield remains unclear.

Microbial communities are closely linked to soil biological functions and may help explain the relationships among biochar addition, SMF, and crop yield [18]. Biochar can reshape microbial community structure by altering soil pH, aeration, carbon availability, moisture, and microsite heterogeneity [19]. However, bacteria and fungi may respond differently to biochar owing to their distinct ecological strategies [19,20]. Bacteria typically exhibit faster growth rates, higher metabolic turnover, and greater sensitivity to labile carbon inputs and nutrient changes [16]. In contrast, fungi possess filamentous hyphal networks that enable greater spatial exploration and the degradation of complex organic substrates [21]. These differences suggest that bacterial and fungal communities may play complementary yet distinct roles in biochar-mediated soil biological functions.

Soil depth may further modulate the microbial pathways through which biochar influences SMF and crop yield [22]. Topsoil, which directly receives biochar amendments, is strongly influenced by root activity, rhizodeposition, fertilizer inputs, and greater oxygen availability; these conditions may favor bacterial growth and bacteria-mediated nutrient turnover [23]. In contrast, the subsoil typically has lower labile carbon availability, reduced rhizodeposition and more heterogeneous nutrient distribution [24]. Under such resource-limited conditions, fungi may be better adapted because their extensive hyphal networks enable them to explore soil pores, connect spatially separated nutrient patches, and degrade complex organic substrates [24]. However, whether the associations of bacterial and fungal communities with SMF and crop yield differ between topsoil and subsoil remains poorly understood, particularly in continuous vegetable cropping systems.

To investigate how biochar influences SMF and eggplant yield, we conducted a field experiment in a continuous eggplant cultivation system on Mollisols in Northeast China. Four biochar application rates were tested to assess their effects on eggplant growth, photosynthetic performance, root development, soil physicochemical and biological properties, microbial community structure, SMF, and yield. We hypothesized that (1) biochar addition would improve eggplant growth and yield in association with changes in soil physicochemical and biological properties; (2) soil biological properties would show stronger associations with SMF and yield than individual nutrient pools; and (3) associations between microbial communities and SMF would vary with soil depth, with bacterial communities more closely associated with SMF in the topsoil and fungal communities more closely associated with SMF in the subsoil.

## 2. Materials and Methods

### 2.1. Experimental Site and Materials

The field experiment was conducted under open-field conditions in Jiamusi City, Heilongjiang Province, China (46°40′ N, 130°27′ E). The region has a mid-temperate continental monsoon climate, with a mean annual sunshine duration of 2319.3 h, a mean annual temperature of 3 °C, a mean annual precipitation of 530 mm, and a frost-free period of approximately 146 days. During the experimental period from May to September 2025, the mean air temperature was 19.4 °C and the cumulative precipitation was 451.5 mm. According to the USDA Soil Taxonomy, the soil is classified as a Mollisol [25]. Prior to the experiment, the physicochemical properties of the 0–20 cm soil layer were as follows: soil organic carbon (SOC), 25.84 g kg^−1^; total nitrogen (TN), 2.01 g kg^−1^; total phosphorus (TP), 0.26 g kg^−1^; total potassium (TK), 2.37 g kg^−1^; and pH of 6.32 (soil: water ratio, 1:5, *w*/*v*). Soil particle-size distribution was determined using a laser diffraction particle-size analyzer (Malvern Panalytical, Malvern, Worcestershire, UK), and particle-size fractions were classified according to the system of the International Society of Soil Science (ISSS). The soil contained 10.1% clay (<2 µm), 65.7% silt (2–20 µm), and 24.2% sand (20–2000 µm). The site had been under continuous eggplant cultivation for more than five years.

The biochar was produced from maize straw by pyrolysis at 450 °C for 1 h under oxygen-limited conditions and was obtained from Liaoning Jinhefu Agricultural Development Co., Ltd. (Anshan, China). The biochar had a pH of 7.25, an electrical conductivity of 1.21 dS m^−1^, and total carbon, TN, TP, and TK contents of 721.8, 12.3, 6.9, and 17.1 g kg^−1^, respectively, corresponding to a C/N ratio of 58.7. The biochar particle size ranged from 1.5 to 2.0 mm. The total pore volume and micropore volume were 0.0074 and 0.0007 mL g^−1^, respectively, and the specific surface area was 1.32 m^2^ g^−1^. The ash content was 16.43%.

### 2.2. Experimental Design

Based on preliminary field experiments conducted by our research group, three biochar application rates were selected to represent increasing input levels within the application range previously evaluated under local field conditions. Accordingly, four biochar treatments were established at application rates of 0 (CK), 5.40 (T1), 6.75 (T2), and 8.10 (T3) Mg ha^−1^. The experiment followed a randomized complete block design with three replicates per treatment (Figure S1). The 12 plots were arranged in a 4 × 3 grid, with each block consisting of four plots aligned in the east–west direction and the three blocks were arranged along the north–south direction (Figure 1). The four treatments were randomly assigned within each block. Each plot measured 5 m × 6 m (30 m^2^). Biochar was uniformly incorporated into the 0–20 cm soil layer before planting, after which raised ridges were prepared.

Greenhouse-raised eggplant seedlings were transplanted into the field in May 2025 in a double-row pattern on raised ridges, with a row spacing of 50 cm and a plant spacing of 35–40 cm. All treatments received identical field management. A compound N-P-K fertilizer was obtained from Harbin Yinong Chemical Fertilizer Co., Ltd. (Harbin, China) and applied uniformly to all plots at rates equivalent to 225 kg N ha^−1^, 120 kg P ha^−1^, and 120 kg K ha^−1^. The fertilizer was applied once as a basal fertilizer before eggplant transplanting and incorporated into the 0–20 cm soil layer. No additional mineral fertilizer was applied during the growing season.

### 2.3. Soil Sampling and Laboratory Analyses

Soil samples were collected from the same experimental plots in July 2025 (flowering stage) and September 2025 (fruiting stage). At each sampling time, soil samples were collected from the 0–20 cm (topsoil) and 20–40 cm (subsoil) layers in each plot using a five-point sampling method. Five soil subsamples collected at the five sampling points from the same depth within each plot were combined to form one composite sample, resulting in one composite sample per plot and soil depth at each sampling time.

After manually removing the visible plant residues, roots, and stones from the sample, place it in a hard container to prevent it from being compressed during transportation. Soil bulk density (BD) was determined separately using the core-ring method. At each sampling time, five undisturbed soil cores were collected from each soil depth in each plot using 100 cm^3^ stainless-steel core rings. The cores were oven-dried at 105 °C to constant weight, and the five measurements were averaged to obtain a plot-level BD value for each soil depth.

In the laboratory, the soil samples were gently homogenized and passed through a 2 mm sieve. Each composite sample was divided into three subsamples: (1) air-dried and sieved for physicochemical analyses, (2) stored at 4 °C for enzyme activity and other biological assays, and (3) stored at −80 °C for microbial community analysis.

SOC and TN were determined using an elemental analyzer [26]. TP was measured by the molybdenum antimony blue colorimetric method following H_2_SO_4_–HClO_4_ digestion [24]. Available phosphorus (AP) was extracted with 0.5 mol L^−1^ NaHCO_3_ and quantified colorimetrically [4]. TK was analyzed by inductively coupled plasma optical emission spectrometry (ICP–OES) after acid digestion [26]. Available potassium (AK) was extracted with 1 mol L^−1^ NH_4_OAc and determined using ICP–OES. Soil pH was measured in a 1:5 (*w*/*v*) soil-to-water suspension. Alkali-hydrolyzable nitrogen (AN) was determined using the alkaline hydrolysis diffusion method [4].

Microbial biomass carbon (MBC) and microbial biomass nitrogen (MBN) were quantified using the chloroform fumigation–K_2_SO_4_ extraction method [27]. Fresh soil samples were divided into fumigated and non-fumigated portions. The fumigated portions were exposed to ethanol-free chloroform vapor, and both portions were extracted with 0.5 mol L^−1^ K_2_SO_4_. MBC and MBN were calculated from the differences in extractable organic C and total N between fumigated and non-fumigated portions.

Soil MBC was calculated as:

MBC = *E_C_*/*k_C_*

where *E_C_* = (organic C extracted from fumigated soils) − (organic C extracted from non-fumigated soils) and *k_C_* = 0.45.

Soil MBN was calculated as:

MBN = *E_N_*/*k_N_*

where *E_N_* = (total N extracted from fumigated soils) − (total N extracted from non-fumigated soils) and *k_N_* = 0.54.

The activities of β-glucosidase (BG), acid phosphatase (ACP), and N-acetyl-β-D-glucosaminidase (NAG) were measured using a microplate fluorometric assay with substrates 4-methylumbelliferyl (MUB)-β-D-glucopyranoside, MUB-phosphate, and MUB-N-acetyl-β-d-glucosaminide as substrates, respectively [16]. Briefly, 1.0 g of fresh soil was suspended in 125 mL of 50 mmol L^−1^ sodium acetate buffer (pH 5.0) and thoroughly homogenized to obtain a homogeneous soil slurry. An aliquot of 200 μL of the soil slurry was transferred into each well of a black 96-well microplate, followed by the addition of 50 μL of the corresponding 200 μmol L^−1^ fluorogenic substrate solution. The microplates were incubated at 25 °C for 4 h in the dark. After incubation, 10 μL of 1.0 mol L^−1^ NaOH was added to each well to terminate the reaction and enhance MUB fluorescence. Fluorescence was measured with a microplate reader at excitation and emission wavelengths of 365 and 450 nm, respectively.

### 2.4. Microbial Community Sequencing and Bioinformatics Analysis

Soil samples collected from all treatments at the fruiting stage were used for amplicon sequencing to characterize bacterial and fungal communities. Total soil DNA was extracted from 0.5 g of fresh soil using a commercial kit (Qbiogene Inc., Carlsbad, CA, USA) following the manufacturer’s protocol. DNA concentration and purity were assessed using a NanoDrop 2000 spectrophotometer (Thermo Fisher Scientific, Waltham, MA, USA). The V3-V4 region of the bacterial 16S rRNA gene was amplified using primers 338F (5′-ACTCCTACGGGAGGCAGCAG-3′) and 806R (5′-GGACTACHVGGGTWTCTAAT-3′). The fungal ITS1 region was amplified using primers ITS1F (5′-CTTGGTCATTTAGAGGAAGTAA-3′) and ITS2R (5′-GCTGCGTTCTTCATCGATGC-3′). For bacterial amplification, each 20 μL PCR mixture contained 4 μL of 5× FastPfu buffer, 2 μL of 2.5 mmol L^−1^ dNTPs, 0.8 μL of each primer (5 μmol L^−1^), 0.4 μL of FastPfu polymerase, 0.2 μL of bovine serum albumin (BSA), approximately 10 ng of template DNA, and nuclease-free water to a final volume of 20 μL. The PCR program consisted of an initial denaturation at 95 °C for 3 min, followed by 27 cycles of denaturation at 95 °C for 30 s, annealing at 55 °C for 30 s, and extension at 72 °C for 45 s, with a final extension at 72 °C for 10 min. For fungal amplification, each 20 μL PCR mixture contained 2 μL of 10× buffer, 2 μL of 2.5 mmol L^−1^ dNTPs, 0.8 μL of each primer (5 μmol L^−1^), 0.2 μL of rTaq polymerase, 0.2 μL of BSA, approximately 10 ng of template DNA, and nuclease-free water to a final volume of 20 μL. The ITS amplification program consisted of an initial denaturation at 95 °C for 3 min, followed by 35 cycles of 95 °C for 30 s, 55 °C for 30 s, and 72 °C for 45 s, with a final extension at 72 °C for 10 min. PCR products were purified, quantified, and pooled in equimolar amounts. Paired-end sequencing was performed on an Illumina MiSeq platform by Majorbio Bio-Pharm Technology Co., Ltd. (Shanghai, China) [28].

Raw reads were quality-filtered using fastp and merged using FLASH based on their overlapping regions. Before downstream analyses, the dataset was rarefied to the minimum number of reads across all soil samples. Sequences were clustered into operational taxonomic units (OTUs) at 97% sequence similarity using UPARSE, which also removed chimeric sequences [29]. Before downstream analyses, the dataset was rarefied to the minimum number of reads across all soil samples. Representative OTU sequences were taxonomically classified using the RDP Classifier against the SILVA database for bacteria and the UNITE database for fungi. The resulting OTU table was used to analyze microbial community composition, microbial diversity, and associations between microbial communities and soil properties [29].

### 2.5. Measurement of Plant Growth, Photosynthetic Characteristics, and Root Traits

Plants with obvious disease symptoms, physical damage, or abnormal growth were excluded according to the same criteria across all plots, after which three healthy eggplant plants were selected from each plot for measurement. Plant height and stem diameter were measured using a measuring tape and a vernier caliper, respectively.

Leaf gas exchange parameters of eggplant leaves, including net photosynthetic rate (Pn), transpiration rate (Tr), and stomatal conductance (Gs), were measured using a portable photosynthesis system (LI-6400, LI-COR Biosciences, Lincoln, NE, USA). Water use efficiency (WUE) was calculated as Pn/Tr. Measurements were conducted between 10:00 and 11:00 on clear days using fully expanded leaves. During measurement, photosynthetically active radiation, reference CO_2_ concentration, leaf-chamber temperature, and air flow rate were maintained at 1000 μmol m^−2^ s^−1^, 400 μmol mol^−1^, 25 °C, and 500 μmol s^−1^, respectively. Root morphological traits were quantified using a root scanner (MES-3200A3L) and WinRHIZO Pro 2019 software (Regent Instruments Inc., Québec, QC, Canada). The same three plants were subsequently used for root measurements. Root systems were carefully excavated with the surrounding soil to minimize the loss of fine roots. The collected roots were gently washed with tap water to remove adhering soil and then rinsed with deionized water. Extraneous plant residues and other debris were manually removed before root analysis. The measured root traits included total root length (RL), total root surface area (RSA), root activity (RA), and total root volume (RV). Fresh root subsamples were immediately collected, and root activity was determined using the 2,3,5-triphenyltetrazolium chloride (TTC) reduction method [30]. Eggplant yield was determined on a per-plant basis at harvest. The fresh fruits from each of the three sampled plants in each plot were collected and weighed separately, and the mean value was used as the plot-level yield.

### 2.6. Statistical Analysis

SMF was calculated using principal component analysis (PCA) based on standardized soil physical, chemical, and biological indicators [31]. Briefly, all variables were first standardized. PCA scores were subsequently computed, and the signs of the eigenvectors were adjusted to ensure that higher scores indicated better soil quality.

Statistical analyses were performed using R version 4.6.0. The normality of model residuals and homogeneity of variance were assessed using the Shapiro–Wilk and Levene tests, respectively. Linear mixed-effects models were fitted with biochar treatment, growth stage, soil depth, and all two-way interactions as fixed effects, and block and plot nested within block as random effects. When significant effects were detected, Tukey-adjusted pairwise comparisons were performed at *p* < 0.05. Different lowercase letters indicate significant differences among treatments. Spearman rank-correlation analysis was used to evaluate relationships among soil properties, microbial diversity, SMF, and eggplant yield. Random forest models were applied to identify the most important predictors of SMF and yield, with variable importance expressed as %IncMSE using the randomForest (v. 4.7-1.2) and rfPermute (v. 2.5.5) packages. Differences in microbial community composition among biochar treatments were further assessed by permutational multivariate analysis of variance (PERMANOVA) based on Bray–Curtis dissimilarities with 999 permutations. Partial least squares path modeling (PLS-PM) was conducted using the plspm package (v. 0.6.0) to assess direct and indirect effects among biochar application rate, soil properties, microbial properties, SMF, and yield. Figures were generated using ggplot2 (v. 4.0.3), vegan (v. 2.7-3), pheatmap (v. 1.0.13), and corrplot (v. 0.95). Data are presented as means ± standard errors, and different lowercase letters indicate significant differences at *p* < 0.05.

## 3. Results

### 3.1. Effects of Biochar Addition on Eggplant Growth and Yield

Biochar addition significantly influenced selected photosynthetic traits, with responses varying between growth stages (Figure 1a). Pn and WUE did not differ significantly among treatments at either growth stage. At the flowering stage, Tr was significantly higher under T2 and T3 than under CK, whereas Gs was significantly higher under T3 than under CK. At the fruiting stage, Tr did not differ significantly among treatments, whereas Gs was significantly higher under T2 than under T1 (Figure 1a). Root morphological traits and root activity also responded to biochar addition (Figure 1b). Overall, T2 and T3 tended to exhibit higher RSA, RV, and root activity than CK, although the magnitude and statistical significance of these responses varied between growth stages. RL exhibited comparatively weak responses to biochar addition, whereas T2 generally exhibited the strongest overall root responses (Figure 1b). Consistent with these belowground responses, treatment effects on aboveground growth became more pronounced at the fruiting stage, with T2 and T3 significantly increasing plant height relative to CK, and T3 producing the greatest stem diameter (Figure 1c,d). Yield showed a similar treatment pattern, increasing by 29.43% under T2 and 18.42% under T3 relative to CK, whereas T1 did not differ significantly from CK.

### 3.2. Effects of Biochar Addition on Soil Physicochemical Properties

More significant treatment effects were observed in the topsoil than in the subsoil (Table S1). In the topsoil, SOC was significantly increased by T2 and T3 at both growth stages, whereas soil pH generally increased with biochar application rates and peaked under T3. T3 also significantly reduced BD and TN relative to CK, whereas AN and AP showed treatment-specific responses; T2 generally exhibited higher values, particularly at the flowering stage (Table S1). In contrast, AK, TP, and TK showed no significant responses to biochar addition. Changes in the subsoil were relatively limited and occurred mainly at the fruiting stage. At this stage, T2 increased AN, whereas T3 showed lower AP and TN than CK (Table S1).

### 3.3. Effects of Biochar Addition on Microbial Biomass and Enzyme Activities

Biochar addition significantly affected soil microbial biomass and extracellular enzyme activities, with responses varying across soil layers and growth stages (Table 1). In the topsoil, T2 generally exhibited the highest MBC and MBN, particularly at the flowering stage, while MBC was the lowest under T3. BG, ACP, and NAG showed no significant treatment responses at the flowering stage, but their responses became more pronounced at the fruiting stage, with T2 generally showing higher enzyme activities and T3 elevating ACP and NAG. In the subsoil, treatment effects were more variable. T3 significantly increased MBC but decreased BG activity relative to CK, while MBN, ACP, and NAG were unaffected. At the fruiting stage, T2 significantly increased MBC, BG, and NAG, whereas T3 resulted in lower MBN and higher NAG than CK. Overall, T2 produced the most consistent increases in microbial biomass and enzyme activities, particularly at the fruiting stage (Table 1).

### 3.4. Soil Multifunctionality and Biological Drivers Underlying Eggplant Yield Formation

Biochar addition significantly affected SMF, with the strongest response consistently observed under T2 (Figure 2a). At the flowering stage, T2 showed the highest SMF, while T1 and T3 showed intermediate values, both of which were significantly higher than that of CK. At the fruiting stage, T2 again exhibited significantly higher SMF than the other treatments, whereas SMF did not differ significantly among CK, T1, and T3 (Figure 2a). SMF was positively correlated with eggplant yield at both growth stages, with R^2^ values of 0.54 and 0.63 at the flowering and fruiting stages, respectively (Figure 2b). These findings indicate that the integrated soil physicochemical and biological status during the fruiting stage was more closely associated with yield formation.

Random forest analysis identified different yield predictors in the topsoil and subsoil at the fruiting stage (Figure 2c,d). In the topsoil, biological properties attained relatively high importance, with BG, NAG, and ACP exhibiting the highest %IncMSE values (Figure 2c). In the subsoil, NAG showed the highest importance, followed by AN and SOC, while MBC and BD showed comparatively lower importance (Figure 2d). Correlation analysis further revealed that yield and SMF were significantly and positively correlated with pH, BG, ACP, and NAG in the topsoil (Figure 2e), whereas in the subsoil, both variables were primarily positively correlated with NAG (Figure 2f). Overall, these analyses indicate that yield and SMF were closely associated with soil biological properties, particularly extracellular enzyme activities.

### 3.5. Microbial Drivers of Biochar-Enhanced Soil Multifunctionality and Yield

Biochar addition significantly altered bacterial and fungal community structures in both the topsoil and subsoil at the fruiting stage (Figure 3a–d). PLS-PM revealed depth-specific relationships among biochar addition, microbial communities, SMF and yield across soil layers (Figure 3e,f). In the topsoil, biochar showed significant path coefficients with both bacterial and fungal communities, while the bacterial community was more strongly associated with SMF. SMF, in turn, showed a significant positive path coefficient with yield (Figure 3e). In the subsoil, the fungal community exhibited a stronger association with SMF, and the path coefficient for the pathway from SMF to yield was also positive (Figure 3f).

Correlation analysis further demonstrated that the relative abundances of dominant bacterial phyla in the topsoil and dominant fungal phyla in the subsoil were closely associated with soil physicochemical properties, enzyme activities, SMF, and yield (Figure 3g,h). Specifically, the relative abundances of *Actinomycetota*, *Bacillota*, *Bacteroidota*, and *Pseudomonadota* in the topsoil were significantly and strongly positively correlated with enzyme activities and SMF (Figure 3g). In the subsoil, the relative abundances of *Basidiomycota* and *Ascomycota* were closely associated with key biological indicators (Figure 3h). Overall, these findings suggest that shifts in microbial community composition contribute to biochar-induced increases in SMF and yield, with topsoil bacteria and subsoil fungi acting as key biological drivers.

## 4. Discussion

### 4.1. Biochar Enhances Eggplant Yield by Improving Biologically Mediated Soil Multifunctionality

In this study, biochar addition significantly affected eggplant growth, selected photosynthetic traits, root development, and yield, with the most consistent positive responses observed at 6.75 Mg ha^−1^ treatment (Figure 1). Compared with CK, eggplant yield increased by 29.43% under 6.75 Mg ha^−1^ and 18.42% under 8.10 Mg ha^−1^, whereas 5.40 Mg ha^−1^ did not significantly affect yield. These findings support the potential of biochar as a soil amendment for improving vegetable productivity in cultivation systems on Mollisol [8,32]. However, the yield response was not proportional to biochar application rate [10]. Although 8.10 Mg ha^−1^ treatment improved several plant and soil variables relative to CK, it did not increase yield compared with 6.75 Mg ha^−1^ and showed weaker responses for some microbial biomass and available nutrient indicators (Table 1). These results indicate that increasing the biochar application rate from 6.75 to 8.10 Mg ha^−1^ provided no additional yield benefit under the conditions tested. Rather, a moderate amount of biochar appeared to provide a more favorable balance among soil physicochemical conditions, nutrient availability, biological activity, and crop performance [33]. Therefore, the agronomic benefits of biochar should be interpreted not only in terms of direct nutrient supply or physical improvement, but also in relation to coordinated changes in multiple soil functions [6,8].

A key finding of this study is that biochar-induced yield improvement was closely associated with enhanced SMF (Figure 2). Because SMF integrates multiple physical, chemical, and biological properties, it provides a more comprehensive measure of soil quality than any individual soil variable [31]. Biochar addition increased SMF, particularly under 6.75 Mg ha^−1^ treatment, and yield was positively correlated with SMF at both growth stages. Notably, this relationship was stronger at the fruiting stage than at the flowering stage, suggesting that soil functional status during the reproductive period was more closely linked to yield formation [33,34]. The fruiting stage is characterized by a high nutrient demand, substantial assimilate allocation to fruits, active root nutrient uptake, and enhanced belowground biological processes [35]. Under these conditions, the capacity of soil to simultaneously support nutrient cycling, microbial activity, organic matter turnover, and root growth becomes particularly important for determining final yield [35]. Thus, the strong SMF–yield relationship at the fruiting stage highlights the important role of integrated soil functioning in fruit expansion and yield accumulation.

Although biochar altered several soil physicochemical properties, our results suggest that yield formation was more closely associated with soil biological functions (Figure 2). Biochar reduced bulk density, increased pH and SOC content, especially in the topsoil, potentially improving soil aeration, root penetration, water retention, and microbial habitat conditions [36]. In this study, biochar addition, notably at the 6.75 Mg ha^−1^ application rate, generally increased BG, ACP, and NAG activities, particularly at the fruiting stage (Table 1). These enzymes are involved in C, P, and N acquisition, respectively, and their increased activities suggest an enhanced capacity for organic substrate decomposition and nutrient mobilization [37]. Enhanced nutrient availability may support photosynthetic activity and assimilate production, thereby contributing to fruit development and yield formation [35]. These relationships suggest that enzyme-mediated C, N, and P cycling may be an important biological process underlying the association of biochar addition with improvements in SMF and crop productivity [36]. The enhancement of soil biological functions by biochar may be attributed to several complementary mechanisms. First, the porous structure and large specific surface area of biochar can provide protective microsites for microorganisms, thereby promoting microbial colonization and alleviating environmental stress on these microbes [38]. Second, biochar-induced increases in soil pH may generate a more favorable environment for microbial growth, enzyme stability, and nutrient transformations, especially in weakly acidic soils [39]. Third, biochar can increase SOC content and indirectly stimulate belowground C inputs by promoting root growth and rhizodeposition [40]. Increases in root length, root surface area, root volume, and root activity under biochar addition may enhance the supply of root-derived substrates to soil microorganisms, thereby stimulating microbial metabolism and enzyme production [41]. In addition, the reduction in bulk density may improve soil aeration and root–microbe interactions [36]. Through these combined effects, biochar may shift the soil system toward greater biological activity, potentially contributing to improved SMF and higher crop yield.

Nevertheless, the weaker and less consistent responses observed for the 8.10 Mg ha^−1^ treatment indicate that increasing the biochar application rate did not necessarily yield additional benefits. Higher biochar application rates can alter soil pH, soil adsorption capacity, and nutrient retention, potentially affecting nutrient availability and microbial nutrient acquisition [39]. In this study, the 8.10 Mg ha^−1^ treatment was associated with lower AN and AP levels in the topsoil, as well as depressed microbial biomass in some soil layers or at certain growth stages (Table 1). These responses may reflect changes in nutrient retention, adsorption, or microbial access to available substrates at this higher biochar application rate [42], although the present dataset does not permit direct distinction among these mechanisms. Changes in soil pH or ionic conditions may also alter microbial community composition without necessarily improving nutrient cycling efficiency [43]. Taken together, these findings indicate that biochar possesses an optimal application rate range, and that moderate biochar addition is more effective in balancing soil physical improvement, nutrient availability, microbial activity, and crop yield.

### 4.2. Depth-Specific Microbial Pathways Underlying Biochar-Enhanced Soil Multifunctionality and Yield

Given the close association between soil biological functions and yield formation, changes in microbial communities may represent a key pathway through which biochar regulates SMF and crop productivity (Figure 3). The divergent responses across soil layers indicate that microbial sensitivity to biochar amendment varies with soil depth [9]. In the topsoil, bacterial communities appeared to exert a pivotal role in linking biochar addition, SMF, and crop yield (Figure 3). This pattern may partly reflect the ecological properties of the topsoil. The topsoil directly receives biochar inputs and is more strongly influenced by root growth, rhizodeposition, gas exchange, and fertilizer-derived nutrient inputs [44,45]. These factors create a dynamic environment with relatively high substrate availability and rapidly changing physicochemical conditions (Table 1). Some bacterial taxa may respond rapidly to changes in labile carbon and nutrient availability [16,26]. Biochar-induced alterations in topsoil habitat conditions may be associated with shifts in the relative abundances of taxa within *Actinomycetota*, *Bacillota*, *Bacteroidota*, and *Pseudomonadota* [46]. The enrichment or functional activation of these bacterial groups may partly explain the enhancement of enzyme-mediated C, N, and P cycling in the topsoil [47,48,49,50]. Meanwhile, biochar-induced improvements in root growth and activity may have elevated inputs of root exudates, mucilage, and sloughed root cells, thereby providing readily metabolizable substrates for bacterial proliferation, extracellular enzyme production, and nutrient turnover in the rhizosphere [51,52]. This interaction among biochar, root activity, and bacterial functioning may have enhanced mineral nutrient supply to roots and sustained photosynthesis, fruit development, and yield formation, particularly during the fruiting stage when crop nutrient demand is high.

In contrast, fungal communities were more strongly associated with SMF than bacterial communities in the subsoil (Figure 3). Compared with the topsoil, the subsoil generally features lower labile C availability, fewer rhizodeposition inputs, and a more spatially restricted nutrient distribution [53]. Under these resource-limited conditions, bacterial activity may be constrained by substrate availability [16,54]. Fungi, however, possess extensive hyphal networks that enable them to explore soil pores and connect spatially separated nutrient patches [54]. Many fungi can also utilize complex organic substrates. These ecological traits may confer on fungi a competitive advantage in the subsoil, where nutrient supply is less dynamic and more dependent on the decomposition of stable organic substrates [24,54].

The association between subsoil fungal communities and SMF may partly be attributed to organic N turnover (Figure 3). NAG participates in the degradation of chitin-derived and other N-containing organic compounds, and fungal biomass and necromass are important sources of chitin-like materials in soil [55]. The association between shifts in fungal communities and NAG activity is consistent with a potential role of fungi in subsoil organic-N turnover, although NAG activity alone cannot establish fungal contributions or net nitrogen mineralization rates. In the subsoil, where inorganic nutrient availability is relatively limited, the capacity to mobilize organic N may be particularly important for sustaining crop nutrient acquisition throughout the reproductive period [22,24]. The contributions of SOC and available N to yield variation in the deeper layer further suggest that fungal-mediated organic matter decomposition and N transformation may support subsoil multifunctionality and indirectly contribute to crop productivity. Biochar may provide colonization surfaces and protective microsites for fungal hyphae, while also altering soil moisture and aeration conditions [18,39]. Although the direct effect of biochar was weaker in the subsoil than in the topsoil, fungal networks may extend its functional influence by linking carbon-rich microsites, organic residues, and root-derived nutrient zones [55]. This may help explain why shifts in fungal communities were more closely associated with SMF in the subsoil. Overall, these findings are consistent with distinct microbial contributions to biochar responses across soil depths, with topsoil bacteria potentially facilitating nutrient turnover and subsoil fungi contributing to organic matter decomposition and organic N mobilization.

### 4.3. Limitations and Future Perspectives

Although this study provides new insights into the relationships among biochar addition, SMF, microbial communities, and eggplant yield, several limitations should be acknowledged. First, the experiment was conducted at a single site using one biochar type, a limited range of application rates, and one cropping system, which may limit the generalizability of the findings. Second, the relatively small sample size limits the robustness of the random forest analysis, which should therefore be considered exploratory despite the use of cross-validation. Third, the observed relationships among microbial communities, SMF, and yield are correlative and cannot confirm definitive causal or mechanistic links. Finally, the long-term effects of biochar remain uncertain. Future studies should therefore include larger, multi-site and long-term experiments with repeated microbial sampling and direct functional measurements to validate these relationships and clarify the underlying mechanisms.

## 5. Conclusions

Biochar addition improved eggplant growth, SMF, and yield, with the moderate application rate (6.75 Mg ha^−1^) producing the greatest yield increase. Eggplant yield was positively correlated with SMF and key soil biological properties, particularly extracellular enzyme activities. Microbial community composition differed significantly across biochar treatments and soil depths, with topsoil bacterial communities and subsoil fungal communities exhibiting stronger associations with SMF and crop yield. Overall, the moderate biochar application rate produced the most consistent positive responses across soil and plant indicators.

## Figures and Tables

**Figure 1:**
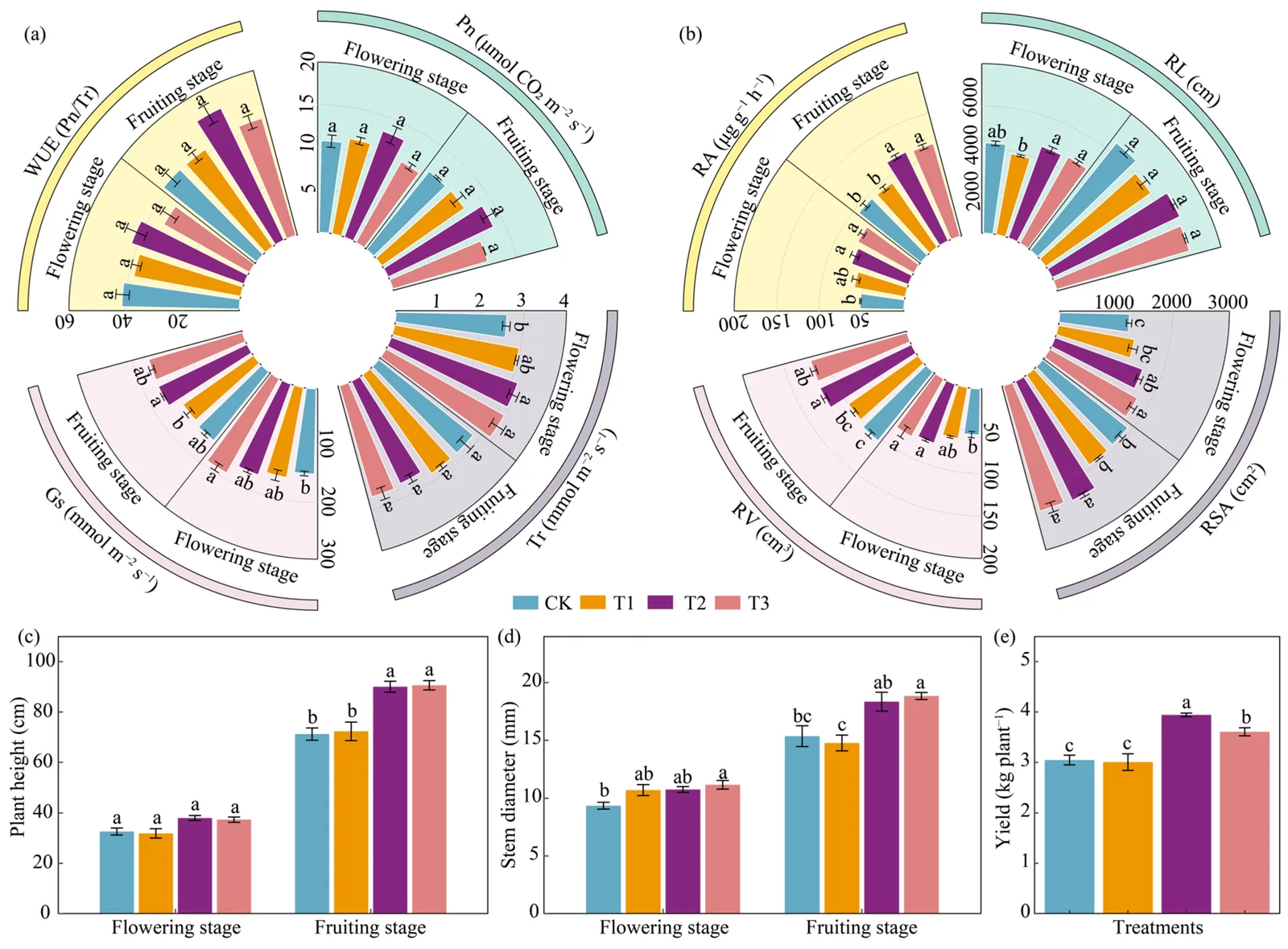
Effects of biochar addition on photosynthetic characteristics, root traits, plant growth, and yield of eggplant. (**a**) Photosynthetic parameters at the flowering and fruiting stages; (**b**) root traits at the flowering and fruiting stages; (**c**) plant height; (**d**) stem diameter; (**e**) yield under different biochar application rates. Pn, net photosynthetic rate; Tr, transpiration rate; Gs, stomatal conductance; WUE, water-use efficiency; RL, root length; RSA, root surface area; RV, root volume; RA, root activity. Data are presented as means ± SE (n = 3). Different lowercase letters indicate significant differences among treatments within the same growth stage and for the parameter at *p* < 0.05. CK, T1, T2, and T3 represent biochar application rates of 0, 5.40, 6.75, and 8.10 Mg ha^−1^, respectively.

**Figure 2:**
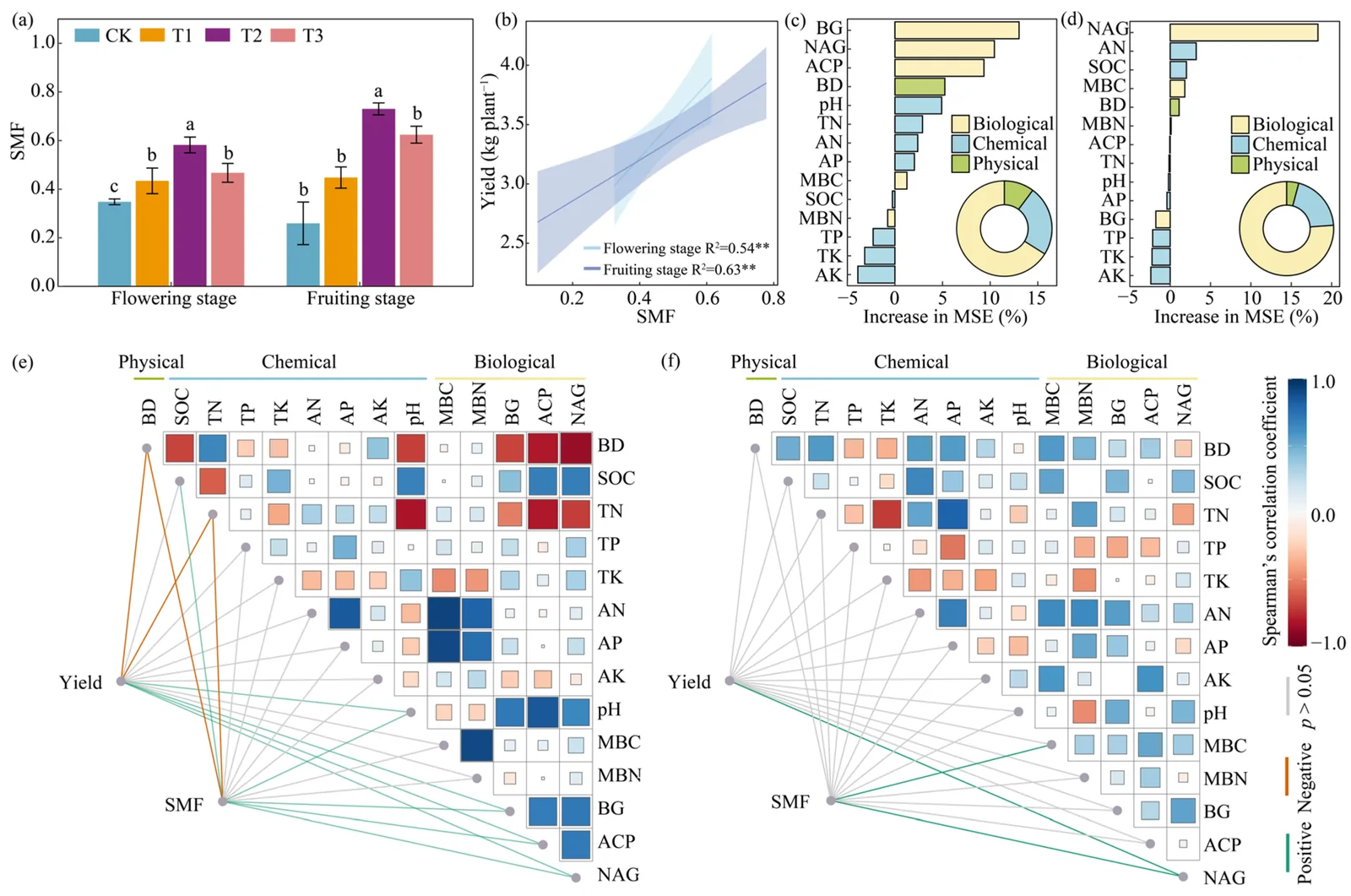
Effects of biochar addition on soil multifunctionality (SMF), yield–SMF relationships, and key soil predictors of eggplant yield. (**a**) SMF under different biochar application rates at the flowering and fruiting stages. (**b**) Relationships between SMF and eggplant yield at the flowering and fruiting stages. The shaded areas represent the 95% confidence intervals. (**c**,**d**) Random forest analysis identifying the relative importance of soil physicochemical and biological properties for yield in the topsoil (**c**) and subsoil (**d**) at the fruiting stage. The inset pie charts show the proportional contributions of physical, chemical, and biological properties. (**e**,**f**) Spearman correlation matrices and correlation networks showing the relationships among soil properties, SMF, and yield in the topsoil (**e**) and subsoil (**f**). Data in (**a**) are shown as means ± SE (n = 3). Different lowercase letters indicate significant differences among treatments within the same growth stage at *p* < 0.05. In (**b**), shaded areas indicate 95% confidence intervals; ** indicates *p* < 0.01. In (**e**,**f**), square color and size represent the direction and strength of Spearman correlation coefficients, respectively; green and orange lines indicate significant positive and negative correlations, respectively, and gray lines indicate non-significant correlations. CK, T1, T2, and T3 represent biochar application rates of 0, 5.40, 6.75, and 8.10 Mg ha^−1^, respectively.

**Figure 3:**
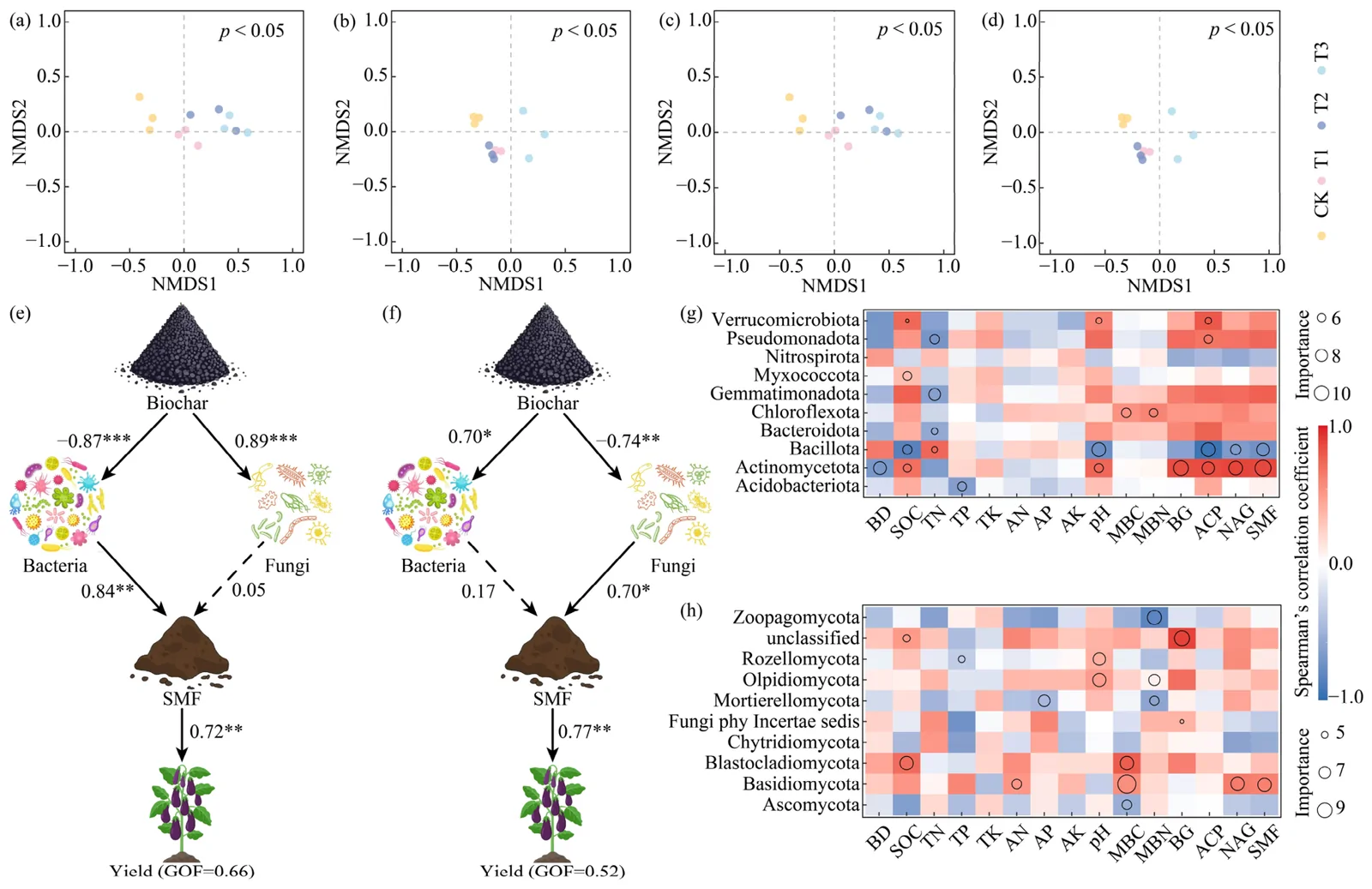
Effects of biochar addition on soil microbial communities and their associations with soil multifunctionality (SMF) and yield. (**a**–**d**) NMDS ordinations of bacterial and fungal communities in the topsoil and subsoil at the fruiting stage. (**e**,**f**) Structural equation models showing the relationships among biochar, bacterial and fungal communities, SMF, and yield in the topsoil and subsoil. respectively. Numbers beside arrows represent standardized path coefficients; solid and dashed arrows indicate significant and non-significant paths, respectively. GOF indicates goodness of fit. Spearman correlations between dominant bacterial (**g**) and fungal (**h**) phyla and soil properties, SMF, and yield. Heatmap colors represent Spearman’s correlation coefficient, and circle size represents the relative importance of microbial taxa. * *p* < 0.05, ** *p* < 0.01, and *** *p* < 0.001. CK, T1, T2, and T3 represent biochar application rates of 0, 5.40, 6.75, and 8.10 Mg ha^−1^, respectively.

**Table 1:** Effects of biochar addition on soil biological properties.

Variables		Flowering Stage				Fruiting Stage			
		CK	T1	T2	T3	CK	T1	T2	T3
0–20 cm	MBC	255.79 ± 8.37 c	276.79 ± 2.97 b	301.75 ± 4.91 a	229.01 ± 4.26 d	261.76 ± 4.67 b	286.79 ± 8.34 a	301.56 ± 4.46 a	240.91 ± 7.86 c
	MBN	33.5 ± 1.51 c	41.47 ± 1.35 b	46.44 ± 2.28 a	39.19 ± 1.12 b	37.99 ± 0.35 b	44.22 ± 2.49 a	45.09 ± 2.18 a	35.6 ± 1.82 b
	BG	67.79 ± 2.96 c	76.61 ± 3.16 b	96.53 ± 1.59 a	92.36 ± 3.51 a	53.85 ± 8.99 b	57.51 ± 2.22 b	80.23 ± 1.11 a	72.42 ± 6.33 a
	ACP	92.39 ± 2.62 a	83.86 ± 1.92 a	84.64 ± 4.72 a	84.12 ± 4.03 a	93.37 ± 3.51 c	119.56 ± 3.89 b	139.34 ± 7.14 a	138.73 ± 7.92 a
	NAG	82.74 ± 7.64 a	85.63 ± 5.15 a	85.88 ± 7.73 a	89.79 ± 20.7 a	100.51 ± 2.32 b	103.97 ± 9.34 b	162.95 ± 18.42 a	153.61 ± 12.95 a
20–40 cm	MBC	244.69 ± 3.75 b	245.56 ± 0.93 b	251.66 ± 5.26 b	266.89 ± 8.18 a	251.82 ± 1.57 c	267.54 ± 5.34 b	287.62 ± 5.37 a	251.64 ± 10.09 c
	MBN	34.24 ± 1.62 a	36.1 ± 1.93 a	38.37 ± 2.83 a	39.01 ± 1.39 a	41.54 ± 1.77 a	41.06 ± 1.43 a	41.54 ± 1.14 a	36.67 ± 1.03 b
	BG	87.25 ± 7.07 a	90.92 ± 3.66 a	93.56 ± 4.49 a	70.59 ± 7.76 b	64.57 ± 3.32 b	53.7 ± 4.56 b	83.72 ± 7.77 a	60.71 ± 6.52 b
	ACP	83.39 ± 1.99 a	94.32 ± 6.77 a	82.52 ± 0.55 a	84.65 ± 8.94 a	106.34 ± 12.98 a	111.73 ± 13.76 a	114.45 ± 6.88 a	99.61 ± 4.05 a
	NAG	60.72 ± 5.59 a	67.53 ± 3.47 a	75.7 ± 12.29 a	67.39 ± 3.64 a	85.41 ± 9.13 b	84.51 ± 4.65 b	122.48 ± 4.68 a	107.75 ± 7.66 a

Note: MBC, microbial biomass carbon (mg kg^−1^); MBN, microbial biomass nitrogen (mg kg^−1^); BG, β-glucosidase activity (nmol g^−1^ soil h^−1^); ACP, acid phosphatase activity (nmol g^−1^ soil h^−1^); NAG, N-acetyl-β-D-glucosaminidase activity (nmol g^−1^ soil h^−1^). Different lowercase letters within the same sampling stage and soil layer indicate significant differences among treatments at *p* < 0.05. CK, T1, T2, and T3 represent biochar application rates of 0, 5.40, 6.75, and 8.10 Mg ha^−1^, respectively.

## Data Availability

The original data presented in the study are openly available in NCBI database at accession codes SUB16455042 and SUB16454850.

## Supplementary Materials

The following supporting information can be downloaded at https://www.mdpi.com/article/10.3390/microorganisms14102139/s1: Figure S1. Schematic layout of the randomized complete block design with four treatments and three replicates. CK, T1, T2, and T3 represent biochar application rates of 0, 5.40, 6.75, and 8.10 Mg ha^−1^, respectively; Table S1. Effects of biochar addition on soil physicochemical properties.
