# Supplementary material for: Biochar Application Improves Eggplant Yield in Association with Changes in Soil Microbial Communities and Soil Multifunctionality
Source: Microorganisms. 2026 Sep 23;14(10):2139. doi: 10.3390/microorganisms14102139 (PMC13707460; doi:10.3390/microorganisms14102139)
Supplement: Supplementary file 1 [file microorganisms-14-02139-s001.zip › microorganisms-4536024-supplementary.pdf]

Biochar Application Improves Eggplant Yield in Association with Changes in Soil Microbial Communities and Soil Multifunctionality

Fangtian Liu †, Qijin Kang †, Chunxiang Song, Yude Wu and Fu Li *

College of Science, Jiamusi University, Jiamusi 154007, China;
13503699906@163.com (F.L.); 19503600219@163.com (Q.K.);
scx09090718@163.com (C.S.); wyd9518@163.com (Y.W.)
* Correspondence: lifu@jmsu.edu.cn
† These authors contributed equally to this work.

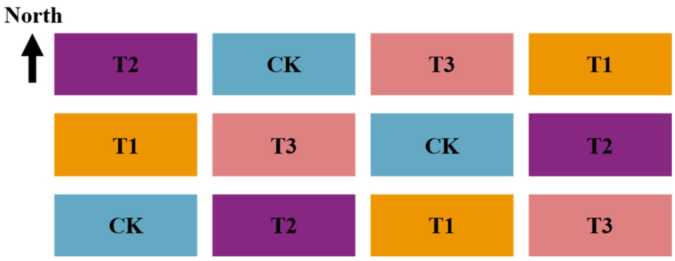

Figure S1 Schematic layout of the randomized complete block design with four treatments and three replicates. CK, T1, T2, and T3 represent biochar application rates of 0, 5.40, 6.75, and 8.10 Mg ha^{-1}, respectively.

Table S1 Effects of biochar addition on soil physicochemical properties

Variables		Flowering stage				Fruiting stage			
		CK	T1	T2	T3	CK	T1	T2	T3
0-20 cm	BD	1.28±0.06a	1.21±0.04ab	1.16±0.03ab	1.1±0.05b	1.4±0.10a	1.33±0.05ab	1.18±0.07ab	1.08±0.13b
	pH	6.16±0.03c	6.21±0.03c	6.37±0.02b	6.57±0.05a	6.15±0.02c	6.27±0.02b	6.33±0.03b	6.54±0.02a
	SOC	25.12±0.88b	27.01±1.21ab	28.5±0.87a	28.98±1.02a	24.59±0.99b	27.64±1.19ab	28.15±1.01a	28.67±1.00a
	AN	106.34±5.56b	118.11±3.75ab	130±3.96a	92.56±2.57c	119.19±3.31b	126.89±3.06ab	131.28±2.52a	92.34±2.78c
	AP	6.57±0.07b	6.59±0.02b	7.77±0.18a	5.44±0.36c	7.07±0.69a	7.51±0.34a	8.11±0.07a	5.15±0.08b
	AK	150.04±6.58a	155.16±15.88a	142.72±18.01a	147.21±11.77a	149.99±7.31a	148.01±13.51a	147.49±13.77a	142.25±14.15a
	TN	2.25±0.12a	2.08±0.08a	2.03±0.10ab	1.76±0.09b	1.94±0.08a	1.85±0.09a	1.72±0.07ab	1.53±0.09b
	TP	0.26±0.02a	0.26±0.01a	0.27±0.01a	0.27±0.03a	0.24±0.04a	0.24±0.02a	0.26±0.02a	0.24±0.01a
	TK	2.57±0.12a	2.39±0.17a	2.49±0.26a	2.55±0.17a	2.45±0.17a	2.52±0.06a	2.49±0.07a	2.71±0.13a
20-40 cm	BD	1.37±0.04a	1.35±0.05a	1.34±0.09a	1.36±0.06a	1.37±0.12a	1.37±0.02a	1.42±0.12a	1.27±0.02a
	pH	5.79±0.11a	5.65±0.11a	5.77±0.22a	5.79±0.02a	5.67±0.07a	5.58±0.04a	5.73±0.09a	5.74±0.03a
	SOC	24.24±0.24a	24.93±1.15a	24.88±1.35a	24.48±0.97a	25.22±1.09a	25.96±0.33a	27.25±0.61a	25.7±0.42a
	AN	105.33±5.05a	115.77±4.01a	112.02±7.41a	112.05±14.26a	118.53±7.9bc	123.42±2.03ab	135.96±4.06a	106.35±4.29c
	AP	6.31±0.17a	6.53±0.15a	6.61±0.16a	6.44±0.56a	7.69±0.83a	6.91±0.58ab	7.31±0.11ab	5.72±0.15b
	AK	154.49±9.65a	148.9±5.12a	150.3±16.46a	146.34±16.25a	143.54±9.03a	152.78±9.76a	155.13±6.36a	145.99±4.62a
	TN	2.04±0.08a	1.95±0.11a	1.99±0.02a	1.93±0.31a	1.95±0.18a	1.83±0.03ab	1.79±0.04ab	1.61±0.04b
	TP	0.26±0.01a	0.27±0.03a	0.26±0.01a	0.24±0.02a	0.22±0.02a	0.28±0.04a	0.25±0.02a	0.26±0.01a
	TK	2.46±0.06a	2.51±0.09a	2.66±0.11a	2.54±0.14a	2.47±0.10a	2.37±0.10a	2.47±0.10a	2.61±0.13a

Note: BD, bulk density (g cm^{-3}); pH, soil pH; SOC, soil organic carbon (g kg^{-1}); AN, available nitrogen (mg kg^{-1}); AP, available phosphorus (mg kg^{-1}); AK, available potassium (mg kg^{-1}); TN, total nitrogen (g kg^{-1}); TP, total phosphorus (g kg^{-1}); TK, total potassium (g kg^{-1}). Different lowercase letters within the same sampling stage and soil layer indicate significant differences among treatments at $p < 0.05$. CK, T1, T2, and T3 represent biochar application rates of 0, 5.40, 6.75, and 8.10 Mg ha^{-1}, respectively.
